# Association of breakfast consumption with body mass index and prevalence of overweight/obesity in a nationally-representative survey of Canadian adults

**DOI:** 10.1186/s12937-016-0151-3

**Published:** 2016-03-31

**Authors:** Susan I. Barr, Loretta DiFrancesco, Victor L. Fulgoni

**Affiliations:** 1 University of British Columbia, 2205 East Mall, Vancouver, V6T 1Z4 Canada; 2 Source! Nutrition, 303-2511 Bloor Street West, Toronto, M6S 5A6 Canada; 3 Nutrition Impact LLC, 9725 D Drive N, Battle Creek, MI 49014-8514 USA

**Keywords:** Breakfast, Overweight and obesity, National survey, Body mass index

## Abstract

**Background:**

This study examined the association of breakfast consumption, and the type of breakfast consumed, with body mass index (BMI; kg/m^2^) and prevalence rates and odds ratios (OR) of overweight/obesity among Canadian adults. These associations were examined by age group and sex.

**Methods:**

We used data from non-pregnant, non-lactating participants aged ≥ 18 years (*n* = 12,377) in the Canadian Community Health Survey Cycle 2.2, a population-based, nationally-representative, cross-sectional study. Height and weight were measured, and BMI was calculated. Breakfast consumption was self-reported during a standardized 24-h recall; individuals were classified as breakfast non-consumers, consumers of breakfasts that included ready-to-eat cereal (RTEC) or as other breakfast consumers. Mean BMI and prevalence and OR of overweight/obesity (BMI ≥ 25) were compared among breakfast groups, with adjustment for sociodemographic variables (including age, sex, race, marital status, food security, language spoken at home, physical activity category, smoking, education level and supplement use).

**Results:**

For the entire sample, mean BMI was significantly lower among RTEC-breakfast consumers than other breakfast consumers (mean ± SE 26.5 ± 0.2 vs. 27.1 ± 0.1 kg/m^2^), but neither group differed significantly from breakfast non-consumers (27.1 ± 0.3 kg/m^2^). Similar results were seen in women only, but BMI of men did not differ by breakfast category. Overweight/obesity prevalence and OR did not differ among breakfast groups for the entire sample or for all men and women separately. When examined by sex and age group, differences were inconsistent, but tended to be more apparent in women than men.

**Conclusion:**

Among Canadian adults, breakfast consumption was not consistently associated with differences in BMI or overweight/obesity prevalence.

## Introduction

It is widely believed that breakfast consumption, versus non-consumption, protects against overweight and obesity [1–3]. Empirical support is provided by a large number of observational studies, summarized in several systematic reviews and meta-analyses [2, 4, 5]. The most comprehensive of these included an analysis of 88 study groups and yielded a pooled odds ratio (OR) of 1.55 (95 % CI: 1.46, 1.65) for the likelihood of being overweight or obese among breakfast non-consumers versus breakfast consumers [2]. There was a tendency for funnel-plot asymmetry (*p* = 0.086), suggesting a possibility of publication bias. Among these studies, many were conducted with children and adolescents, rather than adults. Moreover, relatively few examined variability within a population associated with age, sex or the consumption of different types of breakfasts, although several did assess associations with breakfasts containing or not containing ready-to-eat cereal (RTEC).

There is also an extensive literature indicating that breakfast consumption is associated with improved nutrient intakes and adequacy [6–11]. In a population-representative sample of Canadian adults, we previously observed that breakfast consumers (versus non-consumers) had higher nutrient intakes and a lower prevalence of nutrient inadequacy [9]. We also noted that intakes of several key nutrients were higher (and prevalence of inadequacy lower) in those who consumed breakfasts containing RTEC compared to those who consumed other breakfasts [9]. Thus, using data from adults in the same population-representative sample of Canadians (in which the overall prevalence of overweight/obesity among adults was 59 % [12]), we sought to assess whether breakfast consumption and the type of breakfast consumed (with or without RTEC) were associated with body mass index (BMI; kg/m^2^) and the prevalence rates and OR of overweight/obesity. Further, we examined whether associations varied by age group and sex.

## Methods

### Data source

The present study is a secondary analysis of data collected in the Canadian Community Health Survey, Cycle 2.2 (CCHS 2.2), a cross-sectional, nationally-representative survey conducted by Statistics Canada in 2004 [13, 14]. The target population for CCHS 2.2 represented approximately 98 % of the Canadian population, and included individuals living in private dwellings in the 10 Canadian provinces. The multistage stratified cluster sampling plan was designed to be representative in terms of age, sex, geography and socioeconomic status [13, 14]. Data collection was completed in person by trained interviewers, who received extensive standardized training in all procedures [14]. Survey components included a 24-h dietary recall, a general health questionnaire to assess socio-demographic and lifestyle variables, and measured height and weight (which were used to calculate BMI) [14]. The response rate for the survey was 76.5 %, and the survey weights included a non-response adjustment. Ethical approval for population surveys conducted by Statistics Canada, such as CCHS 2.2, is based on the authority of the Statistics Act of Canada [15].

### Analytical sample

For this analysis, we included data from CCHS 2.2 respondents aged 18 years and above who were not pregnant or lactating, had measured values for height and weight and completed a valid 24-h recall (*n* = 12,337). The 24-h recall was conducted using a modification of the Automated Multiple Pass Method [13, 14]. In the first “pass”, respondents were asked to list all foods and beverages consumed on the day before the survey. Foods and beverages could be listed in any order; there was no requirement to recall foods in a time sequence. Subsequent “passes” obtained additional details about each food item listed, including the amount consumed and what the respondent called the eating occasion (e.g., breakfast, lunch, dinner, a snack). Thus, for our analysis, “breakfast” included any foods or beverages consumed during the 24-h recall at an eating occasion that the respondent identified as breakfast. Those who did not identify any items as being consumed at breakfast were classified as breakfast non-consumers (i.e., "breakfast skippers"). Those who consumed RTEC as a component of breakfast were classified as RTEC breakfast consumers, and those whose breakfasts did not include RTEC were classified as other breakfast consumers. Approval to conduct the analyses reported in this paper was obtained from the Statistics Canada Research Data Centre program [16], project number 11-SSH-UTO-2848.

### Statistical analysis

Statistical Analysis Software (SAS), version 9.2 (Cary, NC) and SUDAAN, version 10.0 (RTI International, Research Triangle Park, NC) were used to analyze the data. SUDAAN was used to create variance estimates and standard errors (SE) of proportions. All analyses were adjusted for the complex CCHS 2.2 sampling design using appropriate sample weights and, when necessary, the MISSUNIT option in SUDAAN was used due to a number of cases with only one stratum within a primary sampling unit. This option then calculates the variance contribution using the difference from the overall mean of the population. Means, percentages and standard errors were obtained using PROC DESCRIPT. Covariate-adjusted mean BMI values were compared among the three breakfast groups using analysis of variance (i.e., using PROC REGRESS). Overweight/obesity prevalence was defined as the proportion with BMI ≥ 25.0, and was compared among the breakfast groups using a *t*-test. Covariates included age, sex, race, household food security (reflecting minimal or no limitations to household food access in the context of financial resource constraint) [17], marital status, language spoken at home, physical activity category, smoking, level of educational attainment and supplement use. A *p* value of < 0.05 (Bonferroni-adjusted *p* < 0.0167) was used to assess significance of differences by breakfast group. Finally, adjusted OR and 95th percentile confidence limits for overweight/obesity were calculated to compare the two groups of breakfast consumers to breakfast non-consumers.

## Results

### Demographic characteristics

As reported previously, the weighted proportions who did not consume breakfast, consumed RTEC breakfasts and consumed other breakfasts were 11, 20 and 69 %, respectively [9]. Significant differences in demographic characteristics were observed among groups (Table 1). Specifically, breakfast non-consumers were younger and less likely to be married or living common-law than the two groups of breakfast consumers. They were the least likely to use dietary supplements and to be food secure, and the most likely to smoke. Breakfast non-consumers were also more likely to be male than other breakfast consumers, but did not differ significantly from RTEC breakfast consumers in that regard. RTEC breakfast consumers were the most likely to use dietary supplements and be food secure, and were more likely than the other two groups to be white. Other breakfast consumers were intermediate in the proportions that smoked, used dietary supplements and were food secure. They were less likely to speak English at home than RTEC breakfast consumers.

**Table 1:** Demographic data for Canadian adults aged ≥ 18 years by breakfast status^d^

Measures	All (*n* = 12,337)	No breakfast (*n* = 1445)	RTEC breakfast (*n* = 2799)	Other breakfast (*n* = 8093)
Age (y)	46.1 ± 0.3	37.6 ± 0.7^a^	48.4 ± 0.6^b^	46.7 ± 0.3^b^
Male (%)	47.4 ± 0.9	53.6 ± 2.5^a^	47.4 ± 1.9^a,b^	46.4 ± 1.1^b^
Married/common law (%)	63.7 ± 0.8	52.7 ± 2.4^a^	61.2 ± 2.0^b^	66.1 ± 0.9^b^
Post-secondary graduate (%)	52.1 ± 0.9	47.5 ± 2.6	51.9 ± 2.0	52.9 ± 1.1
Physically inactive (%)	56.5 ± 0.9	59.2 ± 2.5	52.2 ± 1.9	57.4 ± 1.1
Dietary supplement use (%)	42.5 ± 0.9	33.4 ± 2.3^a^	48.4 ± 2.0^b^	42.1 ± 1.1^c^
Food secure (%)	93.1 ± 0.4	89.2 ± 1.4^a^	95.5 ± 0.7^b^	92.9 ± 0.6^c^
Smoker (%)	21.2 ± 0.7	35.1 ± 2.4^a^	11.2 ± 1.3^b^	22.2 ± 0.9^c^
White (%)	84.2 ± 0.8	78.4 ± 2.4^a^	91.2 ± 1.4^b^	83.0 ± 1.0^a^
English spoken at home (%)	62.2 ± 1.0	70.4 ± 2.6^a,b^	70.0 ± 2.1^a^	58.5 ± 1.2^b^

^a,b,c^Means with different superscripts are significantly different, *p* < 0.05 (Bonferroni-adjusted *p* < 0.0167)

^d^Data are from the Canadian Community Health Survey Cycle 2.2 (2004) and are shown as weighted mean ± SE. No Breakfast = no food or beverages reported as breakfast; RTEC Breakfast = breakfast that included ready-to-eat cereal (RTEC); Other Breakfast = any other type of breakfast

### Body mass index

Mean values for BMI by age group, sex and breakfast status are displayed in Table 2. For the entire sample (both sexes combined), mean BMI was significantly lower among RTEC breakfast consumers than among other breakfast consumers, but neither of these groups differed significantly from breakfast non-consumers. The same pattern of differences was observed among those aged 51–70 years and ≥ 71 years, whereas among adults ≤ 50 years, BMI did not differ by breakfast group. When the sexes were examined separately, no differences by breakfast group were detected for the entire sample of men or for men up to 50 years of age. Among men aged 51–70 years, BMI was significantly lower in RTEC breakfast consumers than breakfast non-consumers, with other breakfast consumers having an intermediate value that did not differ from either of the other two groups. Among men aged ≥ 71 years, BMI was significantly lower in RTEC consumers than in other breakfast consumers. In this age group, mean BMI appeared lowest in breakfast non-consumers, but high variability meant that differences with the other breakfast groups were not significant. Among the entire group of women, BMI was lower in women who consumed RTEC breakfasts versus other breakfasts, with breakfast non-consumers having a value that did not differ from the other groups. This same pattern was observed among women aged ≥ 71 years. In contrast, among women aged 51–70 years, BMI was significantly lower among breakfast non-consumers than among other breakfast consumers, with RTEC breakfast consumers having an intermediate value that did not differ from the other two groups.

**Table 2:** BMI (kg/m^2^) in Canadians by age group, sex and breakfast status^c^

		All (*n* = 12,241)			Male (*n* = 5204)			Female (*n* = 7037)		
		Breakfast group			Breakfast group			Breakfast group		
Age	*n*	None	RTEC	Other	None	RTEC	Other	None	RTEC	Other
		Weighted mean ± standard error								
18–30 y	2947	26.0 ± 0.5	24.7 ± 0.4	25.1 ± 0.2	25.7 ± 0.5	25.1 ± 0.4	25.5 ± 0.3	26.4 ± 0.8	24.2 ± 0.6	24.8 ± 0.3
31–50 y	3129	27.0 ± 0.4	27.2 ± 0.4	27.2 ± 0.2	27.6 ± 0.7	27.7 ± 0.5	27.3 ± 0.2	26.4 ± 0.5	26.7 ± 0.6	27.1 ± 0.4
51–70 y	3618	28.1 ± 0.6^a,b^	27.4 ± 0.3^a^	28.4 ± 0.2^b^	29.9 ± 0.7^a^	27.6 ± 0.4^b^	28.4 ± 0.3^a,b^	26.0 ± 0.7^a^	27.3 ± 0.5^a,b^	28.2 ± 0.3^b^
≥71 y	2547	27.4 ± 0.7^a,b^	26.5 ± 0.2^a^	27.5 ± 0.2^b^	26.0 ± 0.8^a,b^	26.6 ± 0.3^a^	27.5 ± 0.3^b^	27.8 ± 0.8^a,b^	26.5 ± 0.3^a^	27.5 ± 0.3^b^
All ≥ 18 y	12,241	27.1 ± 0.3^a,b^	26.5 ± 0.2^a^	27.1 ± 0.1^b^	27.5 ± 0.4	26.8 ± 0.3	27.2 ± 0.2	26.7 ± 0.4^a,b^	26.1 ± 0.3^a^	27.0 ± 0.3^b^

^a,b^Means within an age group and sex category (all, male, female) that do not share a common superscript letter differ significantly, *p* < 0.05 (Bonferroni-adjusted *p* < 0.0167)

^c^Values are weighted mean ± SE (data from CCHS 2.2). Adjusted for age, sex, race, supplement use, food security, language spoken at home, physical activity category, smoking, education level and marital status. None = no food or beverages reported as breakfast; RTEC Breakfast = breakfast that included ready-to-eat cereal (RTEC); Other Breakfast = any other type of breakfast

### Prevalence of overweight and obesity

**Fig. 1:**
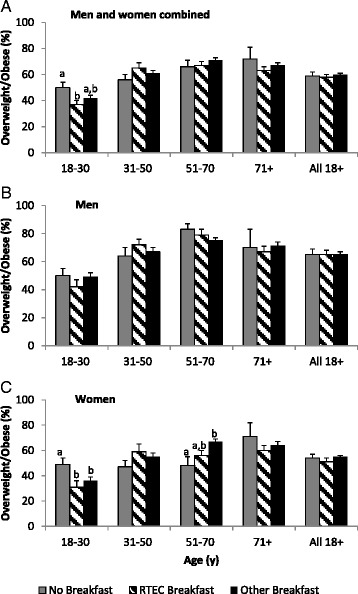
Prevalence (SE) of overweight/obesity (BMI ≥ 25) among Canadian adults, by sex and age group. Values within an age group and sex category (all, male, female) without a common superscript letter differ significantly, *p* < 0.05 (Bonferroni-adjusted *p* < 0.0167). RTEC Breakfast = breakfast that includes ready-to-eat cereal (RTEC). Other Breakfast = any other type of breakfast

The prevalence of overweight and obesity by age group and for adults of all ages is shown in Fig. 1 for both sexes combined and for men and women separately. Overall, no significant differences in overweight/obesity prevalence by breakfast group were seen among adults as a whole, nor were differences observed in all men or all women. When the combined sexes were examined by age group (Panel a in Fig. 1), the only significant difference was among those aged 18–30 years, where overweight/obesity prevalence was significantly higher in breakfast non-consumers than in those who consumed RTEC breakfasts (50 % vs 37 %, respectively). Those who consumed other breakfasts had an intermediate prevalence (42 %) that did not differ from either of the other two groups. No differences were observed in men in any of the age groups (Panel b in Fig. 1). In women (Panel c in Fig. 1), prevalence of overweight/obesity was significantly higher in breakfast non-consumers aged 18–30 years (49 %) than in both groups of breakfast consumers (31 % in RTEC breakfast consumers and 36 % in other breakfast consumers). Conversely, among women aged 51–70 years, the prevalence of overweight/obesity was significantly higher among those who consumed other breakfasts than among breakfast non-consumers (67 % versus 48 %, respectively), while RTEC breakfast consumers had an intermediate prevalence (56 %) that did not differ from either of the other two groups.

Table 3 presents adjusted OR for overweight/obesity for each of the two breakfast groups, compared to breakfast non-consumers (reference group). Data are shown for all adults and for men and women separately, and also for all ages combined and by separate age groups. There were no significant differences in OR for the entire group of adults aged ≥ 18 years, for all men aged ≥18 years or for all women aged ≥ 18 years. When examined by age group, the odds of overweight/obesity were lower among all adults aged 18–30 years who consumed RTEC breakfasts. There were no differences in odds among men of any age group. Among women aged 18–30 years, both groups of breakfast consumers had lower odds of overweight/obesity than breakfast non-consumers, whereas among those aged 51–70 years, consumers of other breakfasts had higher odds of overweight/obesity than non-consumers.

**Table 3:** Odds ratios for BMI ≥ 25 kg/m^2^ by breakfast consumption group among Canadian adults^b^

		No breakfast	RTEC breakfast		Other breakfast	
Age	*n*	Odds ratio (Reference)	Odds ratio	95 % CI	Odds ratio	95 % CI
All						
18–30 y	2947	1.0	0.57	0.36, 0.88^a^	0.72	0.49, 1.05
31–50 y	3129	1.0	1.51	0.91, 2.50	1.22	0.82, 1.84
51–70 y	3618	1.0	1.07	0.62, 1.83	1.28	0.78, 2.08
≥ 71 y	2547	1.0	0.67	0.26, 1.71	0.80	0.31, 2.02
All ≥ 18 y	12,241	1.0	0.95	0.72, 1.26	1.04	0.81, 1.34
Men						
18–30 y	1345	1.0	0.70	0.38, 1.29	0.96	0.56, 1.64
31–50 y	1463	1.0	1.51	0.72, 3.15	1.14	0.60, 2.15
51–70 y	1510	1.0	0.75	0.32, 1.74	0.59	0.30, 1.18
≥ 71 y	886	1.0	0.84	0.24, 1.91	1.01	0.29, 3.53
All ≥ 18 y	5204	1.0	1.03	0.68, 1.57	1.02	0.70, 1.49
Women						
18–30 y	1602	1.0	0.43	0.23, 0.80^a^	0.54	0.34, 0.85^a^
31–50 y	1666	1.0	1.67	0.84, 3.34	1.39	0.83, 2.30
51–70 y	2108	1.0	1.46	0.69, 3.11	2.34	1.17, 4.68^a^
≥ 71 y	1661	1.0	0.58	0.18, 1.81	0.70	0.23, 2.16
All ≥ 18 y	7037	1.0	0.90	0.62, 1.29	1.09	0.80, 1.48

^a^95 % CI for Odds Ratio excludes 1.0

^b^Data from CCHS 2.2. Adjusted for age, sex, physical activity, race, smoking, marital status, supplement use, food security and language spoken at home. Breakfast non-consumers (No Breakfast) were the reference group; RTEC Breakfast = breakfast that included ready-to-eat cereal (RTEC); Other Breakfast = any other type of breakfast

## Discussion

In this population-based study of Canadian adults, neither breakfast consumption (versus non-consumption) nor the type of breakfast consumed (whether or not RTEC was included) was consistently associated with BMI or the prevalence of overweight/obesity. For the overall adult population, mean BMI of breakfast non-consumers and those who consumed other breakfasts was almost identical (27.1 ± 0.3 and 27.1 ± 0.1 kg/m^2^, respectively). While mean BMI of RTEC breakfast consumers (26.5 kg/m^2^) was significantly lower than that of other breakfast consumers, the difference of 0.6 kg/m^2^ reflects a difference of only 1.7 kg at the mean population height of 1.68 m. Furthermore, the prevalence of overweight/obesity and the OR for being overweight/obese did not differ among the three breakfast groups for the adult population as a whole: Overweight/obesity prevalence was close to 60 % in all groups, and adjusted OR (and 95 % CI) for consumers of RTEC breakfasts and other breakfasts were 0.95 (0.72, 1.26) and 1.04 (0.81, 1.34), respectively, relative to breakfast non-consumers. When these associations were examined by sex and age (which has not been done in the majority of previous studies), they were not consistent.

Our results can be compared to those of other population-based, cross-sectional studies of adults [11, 18–25], the majority of which report data on overweight/obesity prevalence. Significantly higher adjusted OR for overweight and/or obesity were observed among all adults who skipped breakfast in studies conducted in Taiwan [18] and Sweden [19], and among both male and female breakfast skippers in a study conducted in Spain [20]. In contrast, OR were not significantly different between breakfast skippers and consumers in studies conducted in Serbia [21] or the United States [22]. Three studies, all conducted in the United States, examined associations with the type of breakfast consumed [11, 22, 23]. One study identified 12 breakfast patterns, including no breakfast [11]. Compared to breakfast non-consumers, the OR for overweight/obesity were lower among consumers of five types of breakfasts, but were similar among consumers of the other six breakfast types (data comparing all breakfast consumers to non-consumers were not provided). In the second study [22], OR for overweight/obesity did not differ between breakfast consumers and non-consumers; however, female but not male consumers of RTEC breakfasts had lower OR for overweight/obesity compared to consumers of other breakfasts. The third study [23] examined young adults aged 20–39 years and found the OR for overweight/obesity was lower in RTEC breakfast consumers, as compared to both breakfast skippers and other breakfast consumers. Results by sex were not reported. Our study is most comparable to the two latter studies [22, 23], in that differences were assessed among those consuming no breakfast, RTEC breakfast and other breakfasts. However, in contrast to Song et al. [22], we found no difference in OR for overweight/obesity between consumers of RTEC and other breakfasts among the entire group of women. And in contrast to Deshmukh-Taskar et al. [23] who studied young adults, in our study the OR was lower among young female consumers of both RTEC and other breakfasts when compared to breakfast skippers.

A smaller number of studies, all of which used data from different waves of the United States National Health and Nutrition Examination Survey (NHANES), report on mean BMI by breakfast intake [11, 23–25]. Cho et al. [24], using data from NHANES III (1998–2004), found that mean BMI was lower among those who consumed RTEC, cooked cereal or quick breads for breakfast than among those who skipped breakfast or consumed breakfast based on meat and eggs. The analysis of Kant et al. [25], with data from NHANES 1999–2004, reported that BMI was lower in women who consumed breakfast, but not in men. Deshmukh-Taskar et al. [23], with data from NHANES 1999–2006, reported that BMI was lower among young adults who consumed RTEC, as compared to breakfast skippers or other breakfast consumers. Finally, the study by O’Neil et al. [11], using data from NHANES 2001–2008, found lower BMI among consumers of four of 11 breakfast types compared to those who did not eat breakfast, but similar BMI among consumers of the other seven breakfast types. We observed a lower BMI among those who consumed RTEC breakfasts compared to those consuming other breakfasts, but neither group differed from breakfast non-consumers.

Variability in the associations between breakfast and weight status also exists within studies. For example, breakfast intake was associated with lower BMI or OR for overweight/obesity in women but not men [22, 25], whereas in another large study [20], the OR for overweight/obesity among breakfast skippers versus consumers were very similar in men and women (1.58 and 1.53, respectively). Our study appears to be the first to examine weight status in association with breakfast by both sex and age group. Although we did detect some differences, for the most part these were observed between consumers of RTEC breakfasts and other breakfasts, rather than between breakfast consumers and non-consumers. The one exception was in women aged 18–30 years, where prevalence and OR for overweight/obesity were lower in both groups of breakfast consumers compared to non-consumers. However, in other age groups there was no evidence for this trend; indeed, among women aged 51–70 years, those who consumed other breakfasts had a significantly higher OR when compared to breakfast non-consumers.

The reasons for different findings across and within studies are difficult to ascertain. Most of the population-based studies described above did not adjust for energy intake [11, 18–21, 24]; in some cases this may have been because data on energy intake were not available [18, 20, 21]. We have previously reported the energy intakes of adult participants in CCHS [9], and although intakes were lower among breakfast non-consumers compared to the two breakfast groups, in the present analysis we chose not to adjust for energy intake as it is on the causal pathway to overweight/obesity. Authors of several other studies have made the same choice [11, 19, 24], while at least one study presented results with energy intake included or excluded as a covariate [22] and others included it [23, 25]. Overall, this adjustment did not appear to differentiate between studies that did or did not detect differences in BMI or obesity prevalence among breakfast groups. Previous studies also differed to some extent in terms of adjusting for other sociodemographic variables, but all controlled for a substantial number of these variables, as did our study. Furthermore, differences in variables that were controlled (e.g., marital status, alcohol consumption) were not consistently associated with study results, suggesting that the extent of statistical adjustment is unlikely to explain the different results. It is possible that cultural differences related to breakfast may play a role, yet studies conducted in countries with different cultures (e.g., Taiwan, Sweden, Spain) [18–20] reported similar findings, and studies conducted in the same country (e.g., the United States) [22, 25] were not always consistent.

Taken together, the lack of consistent patterns of differences in weight status between breakfast consumers and non-consumers, or between consumers of RTEC breakfasts and other breakfasts, appears to argue against a physiologically-based causal relationship. It has been suggested that breakfast consumption may serve as a marker for a healthier lifestyle [3, 26, 27] and that breakfast consumers believe that eating breakfast helps with weight control [3], which may contribute to the associations observed in some studies. To date, the few randomized trials that have been conducted have not provided convincing evidence that breakfast consumption has beneficial effects on weight status [28–30].

It is possible that future research may establish that specific types of breakfast are beneficial for long-term weight management or have other health benefits. For example, among adolescents who habitually skip breakfast, high-protein breakfasts resulted in improved short-term appetite control and satiety [31–33]. Those findings, however, appear to contrast with population-based cross-sectional studies reporting that breakfasts characterized as high in grains and fruit juice, RTEC or cooked cereal were associated with reduced OR of overweight/obesity, whereas breakfasts characterized as high in eggs or meat (and thus higher in protein) were not [11, 24]. Nevertheless, irrespective of whether breakfast itself (or a certain type of breakfast) affects weight status, the overall benefits of breakfast consumption in terms of nutrient intake and diet quality should not be overlooked [6–11].

Strengths of this study include the large population-representative sample, use of measured values for height and weight, examination of associations by sex and age group, and consideration of potentially confounding variables. Limitations are that the data were self-reported and that a single 24-h recall may not reflect habitual patterns of breakfast intake. However, the differences in sociodemographic variables that we observed among breakfast groups suggest that many of those classified in a given breakfast group may have consistently skipped breakfast or consumed a given type of breakfast. We used the conventional BMI cut-point of ≥25 kg/m^2^ to define overweight/obesity, and some research indicates that for older adults, BMI in the overweight range is associated with increased health and longevity [34–37]. We also assessed only two types of breakfasts, and a recent study examined weight status of consumers of 11 different types of breakfasts, as compared to breakfast non-consumers [11]. Nevertheless, like our study, that study also observed variability in the associations between breakfast and weight status, supporting the concept that breakfast per se may not have a consistent impact on weight. Finally, the cross-sectional nature of our data means that causation cannot be inferred. This, however, would be more of a concern if we were reporting strong associations, rather than their absence.

## Conclusions

Among this large population-representative sample of Canadian adults, breakfast consumption was not consistently associated with BMI or overweight/obesity prevalence. Our findings, in conjunction with other observational and experimental evidence, suggest that it may be inappropriate to promote weight-management benefits of breakfast consumption per se. Nevertheless, it is still possible that particular types of breakfast consumption may be helpful for weight management; future long-term randomized trials appear necessary in this regard. In the meantime, the consistently-reported nutritional contributions of breakfast should not be neglected.

## Abbreviations

BMI: body mass index
CCHS: Canadian Community Health Survey
CI: confidence interval
NHANES: National Health and Nutrition Examination Survey
OR: odds ratio
RTEC: ready-to-eat cereal
SE: standard error
y: years

## References

[1] Casazza K, Fontaine FR, Astrup A, Birch LL, Brown AW, Bohan Brown MM (2013). Myths, presumptions, and facts about obesity. N Engl J Med.

[2] Brown AW, Bohan Brown MM, Allison DB (2013). Belief beyond the evidence: using the proposed effect of breakfast on obesity to show 2 practices that distort scientific evidence. Am J Clin Nutr.

[3] Reeves S, Halsey LG, McMeel Y, Huber JW (2013). Breakfast habits, beliefs and measures of health and wellbeing in a nationally representative UK sample. Appetite.

[4] Mesas AE, Munoz-Pareja M, Lopez-Garcia E, Rodriguez-Artalejo F (2012). Selected eating behaviours and excess body weight: a systematic review. Obes Rev.

[5] Horikawa C, Kodama S, Yachi Y, Heianza Y, Hirasawa R, Ibe Y (2011). Skipping breakfast and prevalence of overweight and obesity in Asian and Pacific regions: a meta-analysis. Prev Med.

[6] Williams P (2005). Breakfast and the diets of Australian adults: an analysis of data from the 1995 National Nutrition Survey. Int J Food Sci Nutr.

[7] Deshmukh-Taskar PR, Radcliffe JD, Liu Y, Nicklas TA (2010). Do breakfast skipping and breakfast type affect energy intake, nutrient intake, nutrient adequacy, and diet quality in young adults? NHANES 1999–2001. J Am Coll Nutr.

[8] Gibson SA, Gunn P (2011). What’s for breakfast? Nutritional implications of breakfast habits; insights from the NDNS dietary records. Nutr Bull.

[9] Barr SI, DiFrancesco L, Fulgoni VL (2013). Consumption of breakfast and the type of breakfast consumed are positively associated with nutrient intakes and adequacy of Canadian adults. J Nutr.

[10] Barr SI, DiFrancesco L, Fulgoni VL (2014). Breakfast consumption is positively associated with nutrient adequacy in Canadian children and adolescents. Br J Nutr.

[11] O’Neil CE, Nicklas TA, Fulgoni VL (2014). Nutrient intake, diet quality, and weight/adiposity parameters in breakfast patterns compared with no breakfast in adults: National Health and Nutrition Examination Survey 2001–2008. J Acad Diet Nutr.

[12] Tjepkema M. Nutrition: Findings from the Canadian Community Health Survey. Issue No. 1. Measured Obesity. Adult obesity in Canada: Measured height and weight. Component of Statistics Canada Catalogue no. 82-620-MWE2005001. http://www.statcan.gc.ca/pub/82-620-m/2005001/pdf/4224906-eng.pdf. Accessed 17 Feb 2016.

[13] Health Canada. Canadian Community Health Survey Cycle 2.2 Nutrition, 2004. A guide to accessing and interpreting the data. Health Canada. 2006. http://www.hc-sc.gc.ca/fn-an/surveill/nutrition/commun/cchs_guide_escc-eng.php. Accessed 3 Nov 2015.

[14] Statistics Canada. Canadian Community Health Survey – Nutrition (CCHS), Detailed information for 2004 (Cycle 2.2). Statistics Canada. 2007. http://www23.statcan.gc.ca/imdb/p2SV.pl?Function=getSurvey&Id=7498. Accessed 10 Feb 2016.

[15] Statistics Canada. Statistics Act 1985, c. S-19 amended by 2005, c.38. Statistics Canada. 2005. http://laws-lois.justice.gc.ca/eng/acts/S-19/FullText.html. Accessed 3 Nov 2015.

[16] Statistics Canada. The Research Data Centres (RDC) Program. Statistics Canada. 2014. http://www.statcan.gc.ca/rdc-cdr/index-eng.htm. Accessed 3 Nov 2015.

[17] Health Canada. Canadian Community Health Survey Cycle 2.2 Nutrition (2004). Income-related household food security in Canada. Health Canada 2007. http://www.hc-sc.gc.ca/fn-an/surveill/nutrition/commun/income_food_sec-sec_alim-eng.php#metho25. Accessed 17 Feb 2016.

[18] Huang C-J, Hu H-T, Fan Y-C, Liao Y-M, Tsai PS (2010). Associations of breakfast skipping with obesity and health-related quality of life: evidence from a national survey in Taiwan. Int J Obes.

[19] Berg C, Lappas G, Wolk A, Strandhagen E, Toren K, Rosengren A (2009). Eating patterns and portion size associated with obesity in a Swedish population. Appetite.

[20] Marin-Guerrero AC, Gutierrez-Fisac JL, Guallar-Castillon P, Banegas JR (2008). Eating behaviours and obesity in the adult population of Spain. Br J Nutr.

[21] Grujic V, Cvejin MM, Nikolic EA, Dragnic N, Jovanovic VM, Kvrgic S (2009). Association between obesity and socioeconomic factors and lifestyle. Vojnosanit Pregl.

[22] Song WO, Chun OK, Obayashi A, Cho S, Chung CE (2005). Is consumption of breakfast associated with Body Mass Index in US adults?. J Am Diet Assoc.

[23] Deshmukh-Taskar P, Nicklas TA, Radcliffe JD, O’Neil CE, Liu Y (2013). The relationship of breakfast skipping and type of breakfast consumed with overweight/obesity, abdominal obesity, other cardiometabolic risk factors and the metabolic syndrome in young adults: The National Health and Nutrition Examination Survey (NHANES): 1999–2006. Public Health Nutr.

[24] Cho S, Dietrich M, Brown CJP, Clark CA, Block G (2003). The effect of breakfast type on total daily energy intake and body mass index: Results from the Third National Health and Nutrition Examination Survey (NHANES III). J Am Coll Nutr.

[25] Kant A, Andon MB, Angelopoulos JT, Rippe JM (2008). Association of breakfast energy density with diet quality and body mass index in American adults: National Health and Nutrition Examination Surveys, 1999–2004. Am J Clin Nutr.

[26] Chen J, Cheng J, Liu Y, Tang Y, Sun X, Wang T (2014). Associations between breakfast eating habits and health-promoting lifestyle, suboptimal health status in Southern China: a population based, cross sectional study. J Transl Med.

[27] Keski-Rahkonen A, Kaprio J, Rissanen A, Virkkunen M, Rose RJ (2003). Breakfast skipping and health-compromising behaviors in adolescents and adults. Eur J Clin Nutr.

[28] Dhurandhar EJ, Dawson J, Alcorn A, Larsen LH, Thomas EA, Cardel M (2014). The effectiveness of breakfast recommendations on weight loss: a randomized controlled trial. Am J Clin Nutr.

[29] Schlundt DG, Hill JO, Sbrocco T, Pope-Cordle J, Sharp T (1992). The role of breakfast in the treatment of obesity: a randomized clinical trial. Am J Clin Nutr.

[30] Betts JA, Richardson JD, Chowdhury EA, Holman GD, Tsintzas K, Thompson D (2014). The causal role of breakfast in energy balance and health: a randomized controlled trial in lean adults. Am J Clin Nutr.

[31] Hoertel HA, Will MJ, Leidy HJ (2014). A randomized crossover, pilot study examining the effects of a normal protein vs. high protein breakfast on food cravings and reward signals in overweight/obese “breakfast skipping”, late-adolescent girls. Nutr J.

[32] Leidy JJ, Racki EM (2010). The addition of a protein-rich breakfast and its effects on acute appetite control and food intake in ‘breakfast-skipping’ adolescents. Int J Obes.

[33] Leidy HJ, Ortinau LC, Douglas SM, Hoertel HA (2013). Beneficial effects of a higher-protein breakfast on the appetitive, hormonal, and neural signals controlling energy intake regulation in overweight/obese, “breakfast-skipping”, late-adolescent girls. Am J Clin Nutr.

[34] Flicker L, McCaul KA, Hankey GJ, Jamrozik K, Brown WJ, Byles JE (2010). Body mass index and survival in men and women aged 70 to 75. J Am Geriatr Soc.

[35] Rolland Y, GallinivA CC, Schott A-M, Blain H, Beauchet O (2014). Body-composition predictors of mortality in women aged ≥75 y: data from a large population-based cohort study with a 17-y follow-up. Am J Clin Nutr.

[36] Murayama H, Liang J, Bennett JM, Shaw BA, Botoseneanu A, Kobayashi E (2015). Trajectories of body mass index and their associations with mortality among older Japanese: do they differ from those of Western populations?. Am J Epidemiol.

[37] Veronese N, Cereda E, Solmi M, Fowler SA, Manzato E, Maggi S (2015). Inverse relationship between body mass index and mortality in older nursing home residents: a meta-analysis of 19,538 elderly subjects. Obes Rev.

